# Methane production potential and emission at different water levels in the restored reed wetland of Hangzhou Bay

**DOI:** 10.1371/journal.pone.0185709

**Published:** 2017-10-02

**Authors:** Xuexin Shao, Xuancai Sheng, Ming Wu, Hao Wu, Xiao Ning

**Affiliations:** 1 Wetland Ecosystem Research Station of Hangzhou Bay, Research Institute of Subtropical Forestry, Chinese Academy of Forestry, Hangzhou, Zhejiang, China; 2 East China Forest Inventory and Planning Institute, State Forestry Administration, Hangzhou, Zhejiang, China; Shandong University, CHINA

## Abstract

Changes in the hydrological conditions of coastal wetlands may potentially affect the role of wetlands in the methane (CH_4_) cycle. In this study, the CH_4_ production potential and emissions from restored coastal reed wetlands at different water levels were examined in eastern China at a field scale in two phenological seasons. Results showed that the total CH_4_ flux from reeds at various water levels were positive, indicating that they were “sources” of CH_4_. During the peak growing season, CH_4_ flux from reeds was greater than that during the spring thaw. CH_4_ flux from reeds in inundated conditions was greater than that in non-inundated conditions. The CH_4_ production potential during the peak growing season was far greater than that during the spring thaw. However, the effect of water level on wetland CH_4_ production potential differed among seasons. The correlations among CH_4_ production potential, soil properties and CH_4_ flux change at different water level. These results demonstrate that water level was related to CH_4_ production and CH_4_ flux. The growing season also plays a role in CH_4_ fluxes. Controlling the hydrological environment in restored wetlands has important implications for the maintenance of their function as carbon sinks.

## Introduction

Wetlands are important ecosystems in the context of the global carbon cycle and are the single largest source of atmospheric methane (CH_4_) emissions [1]. Statistically, the CH_4_ content of the atmosphere has increased by 0.8%–1.1% per year in recent years [2]. Wetlands account for 20% of global CH_4_ emissions to the atmosphere [3, 4]. CH_4_ emissions from wetlands are highly variable, both spatially and temporally and at microtopographic to regional scales [5].

CH_4_ emissions that occur in wetlands (natural and constructed) and aquatic ecosystems are the combined result of CH_4_ production, oxidation, and transportation [1, 6]. CH_4_ production is the first step and is a key determinant of CH_4_ emissions. CH_4_ produced under anaerobic conditions is then partly oxidized by methanotrophic bacteria within oxic zones. Three major mechanisms exist that drive CH_4_ transportation: molecular diffusion, bubble ebullition and plant-mediated transportation[1]. Hydrological conditions (e.g., soil moisture content and water level fluctuations) are important ecological characteristics of wetlands. They are among the key factors influencing CH_4_ production in wetlands [7–13]. CH_4_ flux is also negatively affected by salinity so natural salt marshes routinely have lower CH_4_ emissions than do restored wetlands[14, 15]. This is due to substrate competition between sulfate reducing bacteria and methanogens, with less CH_4_ produced in high salinity[7]. Accordingly, much attention has been paid to the factors controlling methanogenesis and CH_4_ emissions from various types of wetlands [16–19] and their contribution to the global warming effect of CH_4_.

Over the last century, around half of the pristine wetland area in countries of the European Union has been drained or subjected to other management practices [20]. At the same time, in China, reclamation of coastal wetland areas has increased rapidly to support economic growth. Currently, 60% of China’s coastline has seawalls. The length of these walls already exceeds that of the historic Great Wall of China [21]. However, to balance wetland protection with economic development, a portion of reclaimed coastal wetlands is often preserved as nearly natural wetlands or replaced with artificial wetlands after development. Thus, human reclamation activities have further facilitated changes in the hydrological conditions of coastal wetland [21, 22], and this has the potential to change the role of wetlands in the global CH_4_ cycle. In reclaimed and restored coastal wetlands, water levels are often controlled by human activities, and CH_4_ emissions are very sensitive to water depth. Existing studies have focused on CH_4_ emissions in natural wetlands, especially freshwater wetlands [7, 9, 23, 24], and have rarely focused on the exchange of greenhouse gases between wetlands and the atmosphere in reclaimed and restored coastal wetlands.

Hangzhou Bay wetland, located at the demarcation line of the northern and southern coastal wetlands of China, is one of the main areas in which coastal wetlands are distributed. Because it is located in the southeast coastal developed areas, it is deeply influenced by human activities. Since China's economic reform, the coastline of south Hangzhou Bay has been pushed back by approximately 16 km in 10 separate instances [25]. In 2005, the Global Environment Fund (GEF) and the World Bank supported the restoration of reclaimed wetlands into swamps, ponds, and shoals using typical aquatic and wetland plants, like reeds (*Phragmites australis*). Research has shown that the presence of the common reed enhances the formation of CH_4_ and also provides a channel for the emission of CH_4_ into the atmosphere [26, 27]. Although restored wetlands cover a much smaller area than pristine wetlands at a global scale, a better understanding of gas exchange especially the impacts of water level on CH_4_ emissions in reclamation wetlands is important because they are amenable to management practices that may help optimize their role in the regional greenhouse gas budget [28]. Therefore, in this study of restored wetlands in Hangzhou Bay, the field CH_4_ gas samples were collected using static chamber for measurements of CH_4_ flux at various water levels. And indoor anaerobic culture methods were used to study the CH_4_ production potential in soil. The relationships between CH_4_ production potential and CH_4_ emissions in restored wetlands were examined to provide a basis for understanding the effects of water level management on the carbon cycle.

## Materials and methods

### Site description

**Fig 1:**
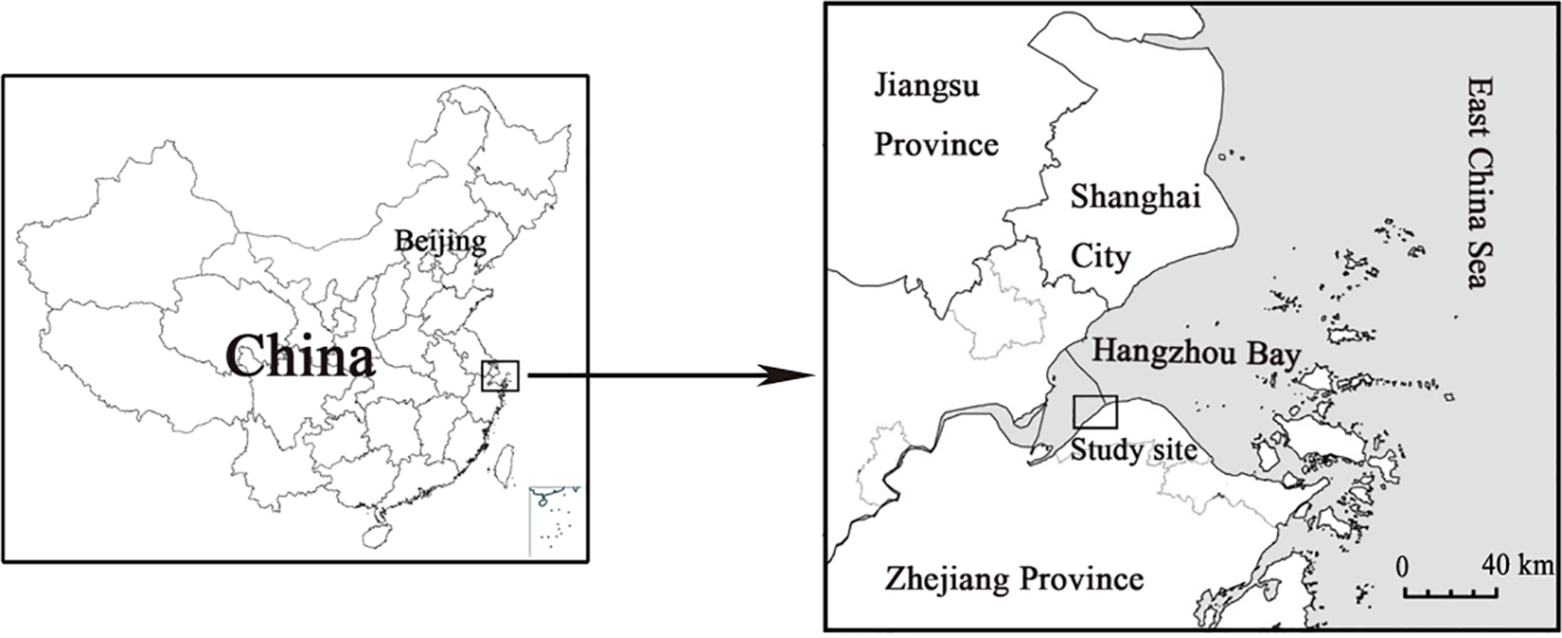
The geographical location of the study area.

Hangzhou Bay is located in northern Zhejiang Province of China (Fig 1). The bay is situated in the outer part of the Qiantang River Estuary, adjacent to the East China Sea, which is one of world's largest tidal bores. Hangzhou Bay is characterized as a north sub-tropical maritime monsoon climate, with four distinct seasons, a mean annual temperature of 16°C and annual rainfall of 1273 mm. The study area was located in a national wetland park (121°09'132″E, 30°19'74″N) in South Hangzhou Bay.

Hangzhou Bay wetlands encompass both natural beaches and restored wetlands. The tidal flat wetlands are greatly influenced by the tides. Monocultures of *P*. *australis* are often found around the high marsh zone, while *Scirpus mariqueter* dominates the low marsh zone. *Spartina alterniflora* often invades the mid-marsh zone and then quickly expands toward the high and low marsh zones [29]. The research zone was located in a restored wetland area. In the zone, at least 80% of the area is swamp, ponds, or shoals. In addition to rainfall, the water can only be supplied by pumping from a River that located outside the wetlands. So the water level is artificially controlled. Around 50% of the water is maintained at a depth of around 30 cm. Reed communities are dominant. They are mainly distributed in shallow and medium depth areas (0–40 cm). Reeds do not typically grow in water deeper than 40 cm.

### Collection and analysis of soil samples

Four areas of restored wetland regions with high percentages of stable vegetation coverage in the Hangzhou Bay were chosen for the study. Each had a different water level, referred to as WL0, WL10, WL20, and WL30. Owing to the combined effects of precipitation and evaporation, the water level in the WL0 zone was basically 0 cm, or roughly even with the ground surface. In the WL10 region, the depth of the water was 5–15 cm and averaged 10 cm. In the WL20 region, the depth was 15–25 cm, with an average of 20 cm. In the WL30 region, the depth of the water was 25–35 cm, with an average of 30 cm. Three 1 m × 1 m quadrats were set for each water level. In order to avoid disturbance to the sampling region and facilitate sampling, wood trestles were built to connect different quadrats. During the spring thaw (April) and peak growing season (September), three replicate soil samples from each of the four water level zones were collected using a Kajak soil sampler (diameter 50 mm) and layered into 0–5 cm, 5–10 cm, and 10–20 cm layers. Each proper amount of soil samples were taken using the quartering method and divided into two parts. About 500 g of soil was refrigerated to the laboratory to measure CH_4_ production potential, while the other part with same weight was freeze-dried first then ground and sieved to determine soil organic carbon (SOC), pH value, redox potential (Eh), and electrical conductivity (EC). Soil pH, Eh, and EC were measured in a 1:2.5 soil:water suspension using a HI2213 pH/ORP/°C Meter (HANNA Instruments, Woonsocket, RI, USA) and DDSJ-308A Conductivity Meter (Shanghai Precision & Scientific Instrument Co., Ltd., Shanghai, China). SOC was measured using a K_2_CrO_7_-H_2_SO_4_ oxidation procedure [30].

### Collection and analysis of CH_4_ gas

The field CH_4_ gas samples were collected using transparent static chamber. During sampling, each quadrat was assigned a static chamber. Five samples were obtained at 10-min intervals during 40-min sampling periods after the chamber was sealed. Detailed structure of the chamber and specific sampling procedures were described by Shao et al. (2017)[31]. A total of 100 ml of sample gas was drawn into the syringe. The sample was maintained in the vacuum bag until the analysis. Sampling was performed in 15^th^-17^th^ in April and 18^th^-20^th^ in September of 2014. Gas sampling was conducted between 08:00 and 10:00 on each sampling day to minimize the influence of diurnal variation in emissions.

The CH_4_ production potential was measured by indoor anaerobic culture methods. Large roots and other extraneous materials were removed and the soil sample was mixed thoroughly until it was uniform. Fresh soil sample (equivalent to 10.0 g dry soil weight) was placed in a 150-ml large-mouth flask with a water to soil ratio of 2.5:1. Water from the sample site was used, and the mixture was mixed thoroughly using a glass rod. The mouth of the flask was sealed with a rubber stopper. There was a hole in the stopper big enough to insert a glass tube with a silicone tube installed on the outside, but small enough to ensure a good seal. This was the sampling tube. All connections used sealant. Next, the flask was evacuated using a vacuum, and the air was replaced with nitrogen. The first vacuum stage was 15 min. Each subsequent vacuum stage was 10 min. The nitrogen was allowed to equalize in the flask for 3–5 min each time. This was repeated 3 times to accomplish a good anaerobic environment. Finally, the vacuum port was sealed and the flask was placed in a dark incubator at 25°C. The first sample was collected 1 day after incubation began. Every day, a small syringe was used to collect a 5-ml sample of gas from the space at the top of the flask for a concentration analysis until the gas concentration stabilized. After each 5-ml sample was collected, 5 ml of N_2_ was injected to equalize the pressure. The CH_4_ production rate was obtained by analyzing changes in the CH_4_ concentration inside the sealed flask over time. Each 5.0-ml sample of gas was obtained from the top part of the flask, and each sample observation lasted for 5 min.

(3) Determination of the CH_4_ concentration

CH_4_ concentration was determined using an Agilent 7890 Gas Chromatograph (Santa Clara, CA, USA) with a flame ionization detector (FID). The chromatographic column was an 80/100 mesh Porapak Q packed column. The column length was 2 m. The carrier gas was N_2_ at a flow rate of 40 ml·min^-1^. The fuel was H_2_ flowing at a rate of 40 ml·min^-1^. The combustion gas was air flowing at a rate of 400 ml·min^-1^. The column temperature was 55°C. The detector temperature was 250°C.

### Data management and analysis

The CH_4_ emission flux was calculated as follows:

$$F=\frac{M}{V}\frac{dc}{dt}H\frac{273}{273+T}$$ (1)

where *F* represents the CH_4_ emission flux mg m-^2^ h^-1^, *M* is the molar mass of CH_4_ in g, *V* is the volume of 1 mol CH_4_ under standard temperature and pressure conditions in L, *dc/dt* is the rate of change of CH_4_ concentration in the static chamber during the sampling period, *H* is the height of the static chamber in m, and *T* is the average temperature inside the static chamber (°C).

The soil’s CH_4_ production potential was calculated using the following equation:

$$P=\frac{\mathrm{dc}}{\mathrm{dt}}\cdot \frac{V_{H}}{W_{S}}\cdot \frac{MW}{MV}\cdot \frac{T_{\mathrm{st}}}{\left(T_{\mathrm{st}}+T\right)}$$ (2)

where *P* indicates the CH_4_ production potential, μg g(dry mass)^-1^ d^-1^; *dc/dt* is the rate of change of gaseous CH_4_ concentration in the flask in mg g^-1^ d^-1^; *V*_H_ is the volume of gas in the upper portion of the flask in L; *W*_S_ is the mass of dry soil in g; MW is the molecular weight of CH_4_ in g; MV is the volume of 1 mol gas under standard temperature and pressure conditions in L; *T* is culture temperature (°C); and *T*_st_ is the standard temperature in K.

The soil CH_4_ flux and production potential under various sampling times or water levels were plotted using Excel 2010 (Microsoft, Redmond, WA, USA). The statistical analysis was implemented in SPSS16.0 (StatSoft Inc. 2007). The two-way analysis of variance (ANOVA) with LSD multiple comparisons was employed to analyze differences in each of the variables among water levels and soil layers at each sampling time. The regression models were used to analyze the relation of CH_4_ production potential to the multiple soil properties and CH_4_ flux. Differences were considered significant if *p*-values were <0.05.

## Results

### Physical and chemical properties of reed wetland soil at various water levels

Tables 1 and 2 summarize the physical and chemical characteristics of the soil during April and September, respectively. Based on ANOVA analysis, for a given season and water level, there were significant differences in physical and chemical properties among soil layers. At WL0, soil pH in the 0–5 cm surface layer was significantly higher than that in deeper soil layers. In inundated conditions (WL10, WL20, and WL30), the pH in the surface soil was significantly lower than that in other layers. The electrical conductivity of soil generally increased with soil depth. At WL0, the soil Eh increased with depth. However, under inundated conditions (WL10, WL20, and WL30) in April, Eh decreased with soil depth. There was no clear Eh gradient under inundated conditions in September. At each water level, the SOC was generally higher in the 0–5 cm surface layer than that in other layers. With respect to the pH at various water levels, in April, the pH was lowest at WL30 and highest at WL0. In September, the opposite results were obtained. The electrical conductivity of soil in April and September was generally higher at WL0 than at other water levels. The soil Eh varied greatly with water level, but the relationship between Eh and depth was not consistent at different water levels. Comparing the SOC at different water levels, for a given soil layer, the SOC was higher at WL0 and WL30 than at WL10 or WL20. The pH and SOC were higher in September than in April, and Eh was lower in September than in April. There was no apparent seasonal difference in the electrical conductivity of soil.

**Table 1:** Basic physical and chemical properties of soil in Hangzhou Bay Wetland in April.

**Water level**		**pH**			Conductivity mS cm^-1^			Eh			SOC g kg^-1^	
	0–5 cm	5–10 cm	10–20 cm	0–5 cm	5–10 cm	10–20 cm	0–5 cm	5–10 cm	10–20 cm	0–5 cm	5–10 cm	10–20 cm
WL0	8.54±0.03(aA)	8.34±0.01(aB)	8.26±0.03(cC)	0.57±0.11(bC)	0.78±0.18(aB)	0.96±0.11(aA)	-97±1.73(cC)	-86±0.58(cB)	-81±1.53(bA)	6.25±0.11(abA)	4.72±0.49(abB)	4.14±0.16(aB)
WL10	8.18±0.03(bC)	8.26±0.01(aB)	8.48±0.04(aA)	0.46±0.18(dB)	0.47±0.05(cB)	0.58±0.14(bA)	-77±1.53(bA)	-87±0.58(cB)	-94±1.00(dC)	5.64±0.13(bA)	4.87±0.13(abB)	4.67±0.30(aB)
WL20	8.05±0.01(cC)	8.23±0.03(bB)	8.42±0.05(bA)	0.53±0.19(cB)	0.50±0.04(bB)	0.61±0.16(bA)	-69±0.58(aA)	-80±1.53(bB)	-91±0.58(cC)	5.86±0.11(bA)	4.25±0.48(bB)	4.03±0.19(aB)
WL30	8.02±0.02(cC)	8.10±0.02(cB)	8.16±0.02(dA)	0.67±0.11(aA)	0.51±0.11(bB)	0.50±0.05(cB)	-67±1.00(aA)	-72±1.00(aBC)	-76±1.00(aC)	7.20±0.43(aA)	5.03±0.15(aB)	4.25±0.37(aC)

Different lowercase letters in brackets indicate significant differences between water levels within a soil layer (*p* < 0.05); different uppercase letters in brackets indicate significant differences between soil layers for the same water level (*p* < 0.05).

**Table 2:** Basic physical and chemical properties of soil in Hangzhou Bay Wetland in September.

Water level		pH			Conductivity mS cm^-1^			Eh			SOC g kg^-1^	
	0–5 cm	5–10 cm	10–20 cm	0–5 cm	5–10 cm	10–20 cm	0–5 cm	5–10 cm	10–20 cm	0–5 cm	5–10 cm	10–20 cm
WL0	8.73±0.03(cA)	8.66±0.01(bB)	8.45±0.03(cC)	0.69±0.11(aC)	0.83±0.18(aB)	0.99±0.11(aA)	-110±1.73(bB)	-104±0.58(aB)	-92±1.53(aA)	7.58±0.11(bA)	7.38±0.49(abB)	6.58±0.16(bC)
WL10	8.65±0.03(dB)	8.98±0.01(aA)	8.98±0.04(aA)	0.41±0.18(cC)	0.49±0.05(cB)	0.60±0.14(bA)	-104±1.53(aA)	-123±0.58(cB)	-128±1.00(cB)	7.37±0.13(bcB)	7.7±0.13(aAB)	6.34±0.30(bcC)
WL20	8.97±0.01(aA)	8.68±0.03(bB)	8.71±0.05(bB)	0.45±0.19(bC)	0.48±0.04(cB)	0.66±0.16(bA)	-121±0.58(cB)	-106±1.53(bA)	-107±0.58(bA)	7.13±0.11(cA)	6.77±0.48(bA)	6.15±0.19(cB)
WL30	8.93±0.02(bA)	8.71±0.02(bB)	8.97±0.02(aA)	0.43±0.11(bcC)	0.53±0.11(bB)	0.58±0.00(bA)	-121±1.00(cB)	-107±1.00(bA)	-122±1.00(cB)	8.44±0.4(aA)	7.69±0.15(aBC)	7.38±0.37(aC)

Different lowercase letters in brackets indicate significant differences between water levels within a soil layer (*p* < 0.05); different uppercase letters in brackets indicate significant differences between soil layers for the same water level (*p* < 0.05)

### Relationship between CH_4_ emission flux and water level in reed wetlands

**Fig 2:**
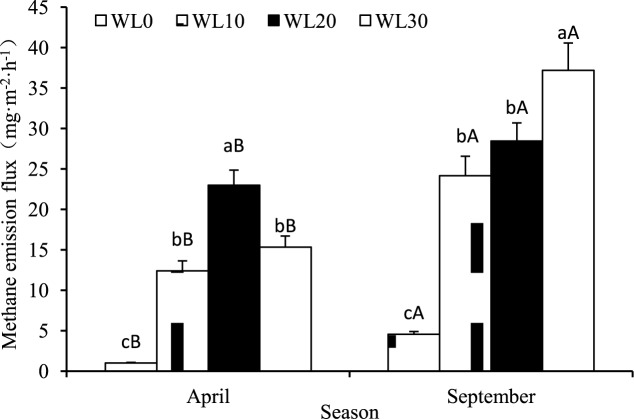
CH_4_ emission flux at various water levels in the Hangzhou Bay Wetland. **(**Different lowercase letters indicate differences between water levels within a season (*p* < 0.05); different upper letters indicate differences between sampling seasons within a water level (*p* < 0.05)).

As shown in Fig 2, at different water levels, reed CH_4_ emissions were consistently positive, indicating that reed wetlands are CH_4_ “source.” In April and September, CH_4_ emission flux varied greatly depending on water level. During the spring thaw, CH_4_ emissions first increased and subsequently decreased as the water level increased. Maximum emissions occurred at WL20. During the growing season, CH_4_ emissions increased gradually with water level. A pairwise analysis of the four water levels showed that during the peak reed growing season (September), CH_4_ emissions were significantly higher than those during the spring thaw (*p* < 0.05). Based on ANOVA analysis, during a given season, CH_4_ emissions in inundated conditions (WL10, WL20, and WL30) were clearly higher than those in WL0 conditions (*p* < 0.05).

### Relationship between CH_4_ production potential and water level in reed wetlands

**Fig 3:**
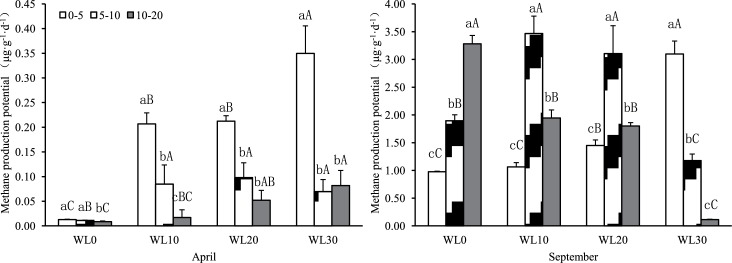
CH_4_ production potential at various water levels in the Hangzhou Bay Wetland. **(**Different lowercase letters indicate differences between soil layers for a particular water level (*p* < 0.05); different upper letters indicate differences between water levels within a soil layer (*p* < 0.05)).

The CH_4_ production potential for 0–20 cm deep soil in reed wetlands in Hangzhou Bay is summarized in Fig 3. During the spring thaw (April) and growing season (September), the maximum values at different water levels occurred on day five of culturing. During the spring thaw (April), the CH_4_ production potential of the 0–5 cm soil layer at different water levels was clearly higher than that of other soil levels. The CH_4_ production potential at 0–20 cm in April decreased according to water level as follows: WL30 (0.167 μg g^-1^ d^-1^) > WL20 (0.121μg g^-1^ d^-1^) > WL10 (0.103μg g^-1^ d^-1^) > WL0 (0.011μg g^-1^ d^-1^). During the growing season (September), at WL0, the 10–20 cm soil layer had the highest CH_4_ production potential. At WL10 and WL20, the 5–10 cm soil layer had the highest CH_4_ production potential. At 0–20 cm, CH_4_ production potential in September was as follows: WL10 (2.158 μg g^-1^ d^-1^) > WL20 (2.118 μg g^-1^ d^-1^) > WL0 (2.050 μg g^-1^ d^-1^) > WL30 (1.462 μg g^-1^ d^-1^). Pairwise comparison indicates that the average CH_4_ production potential for soil in September was clearly higher than that in April.

### Relationships among soil CH_4_ production potential, physical and chemical properties, and CH_4_ emission flux

As shown in Table 3, the CH_4_ production potential of soil at different water levels was positively correlated with SOC and pH. However, the correlation was only significant (*p* < 0.05) at WL10. The CH_4_ production potential of soil was negatively correlated with Eh. However, this correlation was only significant (*p* < 0.05) at WL10 and WL20. There was no significant correlation between CH_4_ production potential and the soil electrical conductivity. Additionally, except at WL30, CH_4_ production potential and CH_4_ emission flux were significantly positively correlated (*p* < 0.05).

**Table 3:** Correlation between soil CH_4_ production potential and soil physical and chemical factors.

*y*	CH_4_ production potential				CH_4_ flux
*x*	SOC	pH	Eh	EC	CH_4_ production potential
WL0	*y* = -2.29+0.545 *x*	*y* = -17.4+2.17 *x*	*y* = -2.19–0.034 *x*	*y* = -2.34+4.16 *x*	*y* = 1.53+1.22 *x*
*R*^*2*^	0.328	0.087	0.079	0.248	0.699*
WL10	*y* = -4.32+0.894 *x*	*y* = -28.7+3.47 *x*	*y* = -4.80–0.058 *x*	*y* = 0.486+1.28 *x*	*y* = 13.9+3.89 *x*
*R*^*2*^	0.687*	0.772*	0.733*	0.005	0.680*
WL20	*y* = -3.09+0.724 *x*	*y* = -19.8+2.46 *x*	*y* = -4.43–0.058 *x*	*y* = 2.42–2.41 *x*	*y* = 23.3+2.18 *x*
*R*^*2*^	0.580	0.468	0.811*	0.025	0.796*
WL30	*y* = -2.47+0.493 *x*	*y* = -12.6+1.58 *x*	*y* = -1.77–0.027 *x*	*y* = 5.47–8.67 *x*	*y* = 21.4+5.95 *x*
*R*^*2*^	0.460	0.334	0.345	0.360	0.352

*indicates significant correlation (*p* < 0.05)

** indicates extremely significant correlation (*p* < 0.01).

## Discussion

### Effects of water level on reed CH_4_ production potential during different seasons

In this study, reed CH_4_ production potential was significantly lower during the spring thaw (April) than during the growing season (September). This is because the reed root systems are relatively undeveloped in April, and SOC and temperature are also low. In the growing season (September), the temperature is relatively higher, roots systems are more developed, and the ability to secrete organic materials is stronger. These conditions provide a rich substrate for the development of methanogenic bacteria and promote CH_4_ production. Thus, the CH_4_ production potential varies among plant growing seasons in accordance with previous findings [32–34].

There were significant differences in the CH_4_ production potential of soil among water levels. In April, the CH_4_ production potential increased as the water level increased. This may be explained by the lower temperatures in April (average air temperature of 16°C and average water temperature of 20°C) than in September. As water level increases, soil temperature also increases. This causes methanogenic bacteria to become more active. Therefore, CH_4_ production potential is higher in areas with deeper water. In September, air temperatures are higher, and soil temperatures in areas of deeper water are low. Except near the surface, CH_4_ production potential decreased with increasing water levels. The 0–20 cm profiles show that the average CH_4_ production potential at WL10 and WL20 was greater than that at WL0 or WL30. These results are in accordance with the results of previous studies. For example, a study on the relationship between CH_4_ production potential and water table fluctuations in two boreal mires indicated that in profiles with constant water levels, the maximal production potential occurs, on average, at approximately 20 cm below the water level [12]. Therefore, the surface water level causes variation in the CH_4_ production potential in different seasons and locations.

Changes in CH_4_ production potential in soil profiles also exhibit significant differences between seasons. In April, the 0–5 cm layer of soil has significantly higher CH_4_ production potential than that of other soil layers at all water levels. This may be explained by the higher soil organic content in the 0–5 cm layer than in other soil layers. In September, the 10–20 cm layer has the highest CH_4_ production potential at WL0. This gradually shifts to the 0–5 cm layer at WL30. This suggests that methanogenic bacteria become more active at relatively shallow soil levels as water levels rise. This observation may be related to the degree of development of reed root systems in different soil layers under different water level conditions and/or rhizosphere properties.

### Effects of physical and chemical soil properties on CH_4_ production potential

Wetland CH_4_ production is a complex biological and geochemical processes. The emission of CH_4_ into the atmosphere is the net result of methanogenesis and CH_4_ oxidation, the rates of which are affected by a number of factors, such as temperature, soil pH, and SOC. It is also affected by CH_4_ production substrates, oxidizers, and the type and quantity of soil microorganisms related to CH_4_ production [13, 14, 23, 35, 36]. Environmental conditions, such as temperature and moisture, were controlled and consistent for all cultures used to examine production potential in this study. Additionally, the SOC was significantly higher during the reed growing season than during the spring thaw. Therefore, it is very likely that differences in production potential are closely related to seasonal variations in SOC [37]. All else being equal, CH_4_ emission flux and SOC are significantly correlated [38]. Liu et al. have shown that substrate and/or substrate-driven changes in the abundance of methanogenic archaea cause seasonal variation in CH_4_ production potential in species-specific freshwater wetlands [16]. In this study, soil pH and CH_4_ production potential were not significantly correlated, except at WL10. Generally, methanogenic bacteria only thrive in a narrow pH band of 6–8. In the study zone, the pH values varied a narrow range with 8.08–8.34 in April and 8.56–8.92 in September. Since pH values were above the suitable range for methanogenic bacteria, their activity was suppressed. This contributed to weakening the correlation. Soil Eh is a measure of the aerobic or anaerobic conditions of the soil, and thereby is related to the rate of CH_4_ production. In this study, except for WL0 and WL30, soil Eh and soil CH_4_ production potential were significantly negatively correlated. This is consistent with the results obtained by Liu et al. in a study of rice fields [39] and indicates that Eh is an important factor affecting CH_4_ production potential [40]. In this study, the electrical conductivity of the soil was not significantly correlated with CH_4_ production potential during either the spring thaw or the growing season. This is because the electrical conductivity of the soil at different water levels did not differ substantially. Therefore, its correlation with CH_4_ production was weak.

### CH_4_ emission flux in reed wetlands at different water levels and its relationship with CH_4_ production potential

In this study, water level has a significant effect on CH_4_ emission flux. The aerobic and anaerobic conditions of the soil, controlled by the water level, are a prerequisite for the production of greenhouse gases. For example, inundation significantly reduces redox potential in soil, which in turn increases CH_4_ production, the CH_4_ concentration of pore water, and CH_4_ emissions [36]. Therefore, CH_4_ emissions in the study zone at WL0 (non-inundated) were always lower than those at other water levels. These results show that there is seasonal variation in the relationship between water level changes and CH_4_ emissions from reed wetlands. During the growing season, as the water level increased, the rate of CH_4_ emission gradually increased, in agreement with previous findings [41, 42]. This may be because reeds in 20–40 cm deep water are tallest and have the largest mass. In the summer, plant photosynthesis is at its peak and ventilation systems are fully mature. Gas “channels” are plentiful and plants can provide a substrate for the production of CH_4_ and a pathway to deliver it to the atmosphere [41, 43]. However, the relationship between CH_4_ emission flux and water level during the spring is initially positive and become negative as the water levels continue to rise. This may be because less of the CH_4_ produced in the soil during the spring thaw is transported by reeds and more finds its way to the atmosphere via water. In this process, CH_4_ can be oxidized to become carbon dioxide. Since water depth increases the opportunity for CH_4_ to be oxidized, less CH_4_ is emitted into the atmosphere [44]. In addition, during the spring thaw, lower water levels are associated with lower soil temperatures. Methanogenic bacteria are inhibited by low temperatures; accordingly, CH_4_ emission flux is lower in areas with shallow water [45].

We found a positive relationship between CH_4_ production potential and CH_4_ emission flux of the soil samples in the study zone. CH_4_ emission is the combined result of CH_4_ production, oxidation, and transport [1]. In wetlands, CH_4_ is mainly delivered to the atmosphere via soil, water, and plant systems. Plants are the main route by which CH_4_ is delivered to the atmosphere in areas with vegetation [11, 26]. In this study, at WL30, CH_4_ production potential and CH_4_ emission flux were not significantly correlated. This may be because in non-inundated areas or areas with low water levels, CH_4_ emitted from the soil is transported directly to the atmosphere by reeds, while in areas with deeper water, the CH_4_ is first transported into the water by plants and then disperses via the water into the atmosphere. This weakens the relationship between CH_4_ production potential and CH_4_ emission flux.

## Conclusions

In this study, changes in CH_4_ production potential and emission in soil at different depths were examined at various water levels in coastal reclamation areas with restored reed wetlands during different seasons. In summary, water level has a significant effect on CH_4_ emission flux and CH_4_ production potential, and these effects differed among growing seasons. In restored coastal wetland areas, CH_4_ production potential and emission flux can be controlled by lowering the water level during the peak reed growing season. This can lower the loss of soil organic carbon in reclamation areas, and promote their function as better carbon sinks.

### Ethics statement

We carried out the study at Wetland Ecosystem Research Station of Hangzhou Bay, which belongs to the Research Institute of Subtropical Forestry, Chinese Academy of Forestry. All necessary permits were obtained for the described field study. This work is unrelated to an ethics issues, and we also confirmed that the field studies did not involve endangered or protected species.

## Supporting information

S1 Table Soil physical and chemical properties, CH4 production potential and emission flux of reed wetland at various water levels. (XLS)
